# Dynamic Analysis of Changes of Protein Levels and Selected Biochemical Indices in Rat Serum in the Course of Experimental Pleurisy

**DOI:** 10.1007/s10753-016-0339-x

**Published:** 2016-04-15

**Authors:** Ireneusz Całkosiński, Jacek Majda, Grzegorz Terlecki, Kinga Gostomska-Pampuch, Katarzyna Małolepsza-Jarmołowska, Sylwia Sobolewska, Aleksandra Całkosińska, Aleksandra Kumala, Andrzej Gamian

**Affiliations:** Independent Laboratory of Neurotoxicology and Environmental Diagnostics, Wroclaw Medical University, Bartla 5, 51-618 Wroclaw, Poland; Department of Laboratory Diagnostics, 4th Military Hospital, Weigla 5, 50-981 Wroclaw, Poland; Department of Medical Biochemistry, Wroclaw Medical University, Chałubińskiego 10, 50-368 Wroclaw, Poland; Institute of Immunology and Experimental Therapy, Polish Academy of Sciences, Weigla 12, 53-114 Wroclaw, Poland; Department of Drugs Form Technology, Wroclaw Medical University, Borowska 211A, 50-556 Wroclaw, Poland; Department of Animal Nutrition and Feed Management, Wroclaw University of Environmental and Life Sciences, Chełmońskiego 38c, 51-630 Wroclaw, Poland

**Keywords:** pleurisy, inflammatory reaction, electrophoresis of serum proteins, acute-phase proteins

## Abstract

A significant role is played in inflammation by the liver, which, stimulated by inflammatory mediators, synthetizes plasma proteins with various dynamics. The purpose of these studies is to generate a detailed dynamic analysis of changes to concentrations of plasma and serum protein fractions and selected acute-phase proteins as well as nonspecific biochemical indices during the course of an induced pleurisy. The studies were conducted on female inbred Buffalo rats, which were divided into two groups: a control group (C) and an experimental group (IP) in which pleurisy was induced. In the IP group, significant changes in biochemical indices were observed between the 48th and 96th hours of pleurisy. A reduction of albumin, transferrin, urea, and creatinine concentrations was observed, while concentrations of the complement components C3 and C4, haptoglobin, and fibrinogen increased. An early increase of IL-1 was observed, while increases of IL-6 and TNF were noted in the later period. The maximum intensity of the processes described above occurred between the 72nd and 96th hours of pleurisy.

## INTRODUCTION

Inflammatory reactions are characterized by the occurrence of numerous subsequent phases which are not possible to diagnose at the initial stage using the diagnostic laboratory methods generally applied at present. However, the most recent studies have proven that their dynamics can be assessed using thermal vision [1]. In inflammatory reactions, there is an acute phase which lasts tens of seconds, from the moment of stimuli activation up to 12 h, which transforms into a chronic inflammation phase. At the first stage of an inflammatory reaction and following the activation of some factors, a reflectory contraction phase of local blood vessels occurs, which is related to the neurogenic response that results from stimulation of pain receptors. Stimulation of these receptors releases a somatic-vegetative impulse, appearing within several seconds of the activation of strong stimuli by releasing catecholamines (adrenaline and noradrenaline) from the adrenals [2]. Its purpose is to reduce bleeding, which has been proven by observations conducted using thermal vision [1]. It also prevents the products of tissue damage which appear most rapidly, such as proteolytic enzymes, as well as shock bodies, such as histamine, from spreading throughout the body. The impact on pain receptors by various mediators (kinins) released by the inflammatory factor leads to a visceral and neurohumoral reaction, which is manifested by increased concentrations of catecholamines and then adrenal glucocorticoids [3].

After a period of reduced blood supply, a local decrease in vascular resistance occurs as a result of histamine release, while, at a later stage, kinins appear, which cause changes in vascular permeability. This leads to swelling as well as a local increase of temperature which can be observed thermovisually [1]. At a later stage of an inflammatory reaction, a significant role is played by the synthesis of acute-phase proteins and proteins of the coagulation cascade taking place in the liver as well as by the increased proteolysis of muscle proteins and fever. This results from the external and internal pyrogens impacting the thermoregulatory center [4–6].

The first stage of the inflammatory reaction described above, related to the release of inflammatory mediators such as histamine, bradykinin, serotonin, and the occurrence of a pain reaction, is difficult to diagnose using laboratory tests. At this stage, no shifts in the leukocyte image nor changes in the image of red blood cells and thrombocytes can be observed. In the first hours of inflammation, there are no significant changes in blood biochemical indices, such as the concentration of acute-phase proteins [7–10].

During inflammation, a significant increase of concentrations of such cytokines as interleukin (IL)-1, IL-6, tumor necrosis factor (TNF)-α, and TNF-γ is observed. They function as regulators of gene expression [11–13]. Inflammation stimulates leukocytes, monocytes, and macrophages to produce acute-phase mediators [14, 15]. These, in turn, stimulate hepatocytes to produce acute-phase proteins. Their increased concentration is responsible for the activation of various processes, such as facilitating phagocytosis of macrophages and microphages.

The initiated inflammatory process and the release of hormones accompanying this phenomenon impact liver metabolism, which itself impacts coagulation processes, activation of the complement system, and changes in the concentrations of metals such as Fe, Cu, and Zn. The latter are associated with increased synthesis of acute-phase proteins, which play a protective role in the management of these metals [7, 8].

Only after several hours of inflammatory reactions it is possible to observe changes in some blood biochemical parameters. Sometimes, they are interpreted incorrectly, due to the lack of detailed analysis regarding the dynamics of changes in concentrations of plasma and serum proteins from the initiation of the inflammatory process to the moment of the examination. At this time, the erythrocyte sedimentation rate (ESR) increases, while the concentrations of numerous plasma proteins change.

**Fig. 1:**
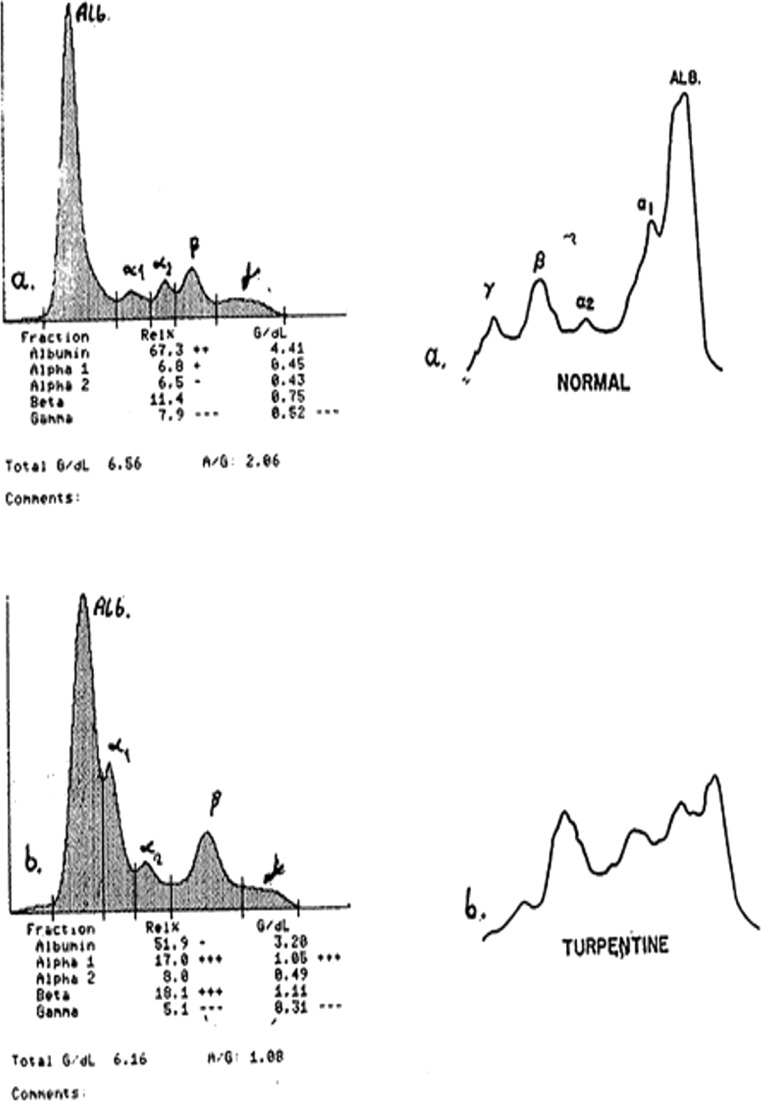
Electrophoresis of rat serum proteins in own study (*I*) [7] and by Weimer (*II*) [32]: *Ia* control, *Ib* carrageenan-induced inflammation (72 h); *IIa* control, *IIb* turpentine-induced inflammation.

Electrophoretic separation of plasma proteins allows us to distinguish the following groups of proteins [16–19] (Fig. 1): albumins, pre-albumins, and globulins, which can be divided into the following fractions: α_1_-globulins which include *inter alia* α_1_-3,5-glycoprotein, α_1_-antitrypsin, and coagulation factors VII, VIII, and IX; α_2_-globulins which include α_2_-macroglobulin and haptoglobin; β-globulins which include transferrin, alanine aminotransferase, fibronectin, and complement system; and γ-globulins which include five classes of immunoglobulins: IgG, IgA, IgM, IgD, and IgE.

Some proteins which belong to the fractions mentioned above are classified in accordance with Koj’s division [20–23] as acute-phase proteins. These proteins are mainly produced in parenchymal liver cells. This division includes the following: group A proteins, consisting of very active proteins whose concentration in the plasma increases 20–100 times; group B proteins, defined as strongly reacting proteins whose concentration increases 2–5 times in the plasma; group C proteins, whose concentration increases 30–60 %; group D proteins, which do not show any significant changes in concentration; and group E proteins, characterized by a decreased concentration in the plasma of 30–60 % of the physiological value [24]. Lebreton *et al.* [25] also proposed the introduction of negative proteins, whose concentration in the plasma decreases during the course of an inflammatory reaction.

Only a few studies of the dynamics of changes to protein levels during a long-term inflammatory reaction using electrophoretic separation of proteins have been conducted. This inspired us to undertake the studies whose results we present in this paper, and during which, we used the electrophoresis technique. The purpose of this study is to provide a detailed analysis of the composition of proteins in dynamic decomposition during a long-term induced inflammatory process of pleurisy.

## MATERIALS AND METHODS

### Experimental Animals

All experiments with the use of animals were approved by the Local Bioethics Council for Animal Experiments (permission number: 23/2001).

The studies were conducted on inbred Buffalo female rats with a body mass of 120–140 g, aged 8–10 weeks. The experiments included animals from a narrow age group and weight group, of the same sex, and with a large kinship coefficient, characterized by very similar reactivity to the inflammatory factor, and the obtained results demonstrated small standard deviations. The animals were bred by the Department of Pathological Anatomy at Wroclaw Medical University. During the experiment, the rats were kept in identical conditions and placed in polystyrene cages with metal covers, six rats in each cage. The experiments were conducted in air-conditioned rooms with a temperature of 21–22 °C and ambient humidity of 62–63 %. The rats were fed with standard *Murigran* feed, and they received water *ad libitum*.

### Initial Examinations of Reactions to Inflammation

Analysis of the obtained results regarding reactions to experimental inflammations of the pleura, peritoneum, and hind limb (feet) allowed us to state that pleurisy is the most authoritative model of inflammatory reaction in respect to the selected hematological and biochemical indices [26].

### Inducing Pleurisy

The animals used for the experiments were divided into the following groups:

1. Control group (C)
2. Experimental group (IP), in which experimental pleurisy was induced by administering 0.15 ml of a 1 % solution of carrageenan (Sigma, USA) into the pleura to the fourth to fifth intercostal space on the right. Carrageenan, as a sulfate polysaccharide extracted from the algae *Chondrus crispus* [27], was diluted in 0.9 % NaCl (Polfa, Poland) before the experiment.

The research material (blood) was collected from the rats under anesthesia induced by pentobarbital (30 mg/kg b.w.) into the peritoneum. Then, the abdominal cavity was opened and a cannula catheter (2 mm diameter) was injected into the aorta, allowing the blood to be collected in standardized hematological and serological test tubes (Sarstedt, Germany).

### Biochemical Assays

High-voltage electrophoresis (100 V, 35 min) of serum proteins (SPE) was conducted on a buffered agarose gel using an analyzer manufactured by Beckman Coulter (USA). Readings and analysis of the results were conducted using a DT 93 densitometer manufactured by Beckman Coulter with a 600-nm wave, using its native software.

Biochemical tests of the blood serum were conducted using a RA-1000 analyzer manufactured by Technikon and reagents produced by Technikon S.A. (Tournai, Belgium). The following components were marked:

- Total protein (TP)—by Weichselbaum’s colorimetric method in Skeggs and Hochstrasser’s modification based on a biuret reaction in an alkaline environment; absorbance photometric measurement with a 550-nm wave; TP concentration was provided in grams per deciliter; the total precision of the test is ≤2.1 % coefficient of variation (CV), and sensitivity is 1.0 g/l;
- Albumin—by Rodkey’s colorimetric method in the modification of Dumasa *et al.*, using bromocresol green in an acidic environment; absorbance photometric measurement with a 600-nm wave; albumin concentration was provided in grams per deciliter; the total precision of the test is ≤1.4 % CV, and sensitivity is 1.0 g/l;
- Urea—by Tolk and Schubert’s method in Tiffany’s modification with urease and glutamate dehydrogenase, using Warburg’s optical test from NADH with a 340-nm wave; urea concentration was provided in milligrams per deciliter; the total precision of the test is ≤2.8 % CV, and sensitivity is 1.1 mmol/l;
- Creatinine—by Jaffy’s colorimetric method in the modification of Chasson *et al.*, with picric acid in an alkaline environment; measuring absorbance with a 500-nm wave; creatinine concentration was provided in milligrams per deciliter; the total precision of the test is ≤1.7 % CV, and sensitivity is 2 μmol/l;
- Aspartate aminotransferase (AST)—by Karmen’s method modified by Bergmeyer and recommended by the International Federation of Clinical Chemistry (IFCC), in a Tris-HCl buffer, with l-aspartate and pyridoxal phosphate, using an optical test from NADH, with a 340-nm wave at a temperature of 37 °C (310 K); AST activity was provided in units per liter; the total precision of the test is ≤2.0 % CV, and sensitivity is 2.0 U/l;
- Alanine aminotransferase (ALT)—by Wróblewski and LaDue’s kinetic method modified by Bergmayer as recommended by the IFCC in a Tris-HCl buffer, with l-alanine and pyridoxal phosphate, using an optical test from NADH, with a 340-nm wave at a temperature of 37 °C (310 K); ALT activity was provided in units per liter; the total precision of the test is ≤2.5 % CV, and sensitivity is 2.0 U/l.

### Acute-Phase Protein Assays

Acute-phase proteins were marked on an analyzer manufactured by Technikon RA-1000 System:

- C3 complement components (mg/dl) and C4 complement components (mg/dl) were marked by an immunoturbidimetric method; the sensitivity of the method is 0.02 and 0.001 g/l for C3 and C4, respectively;
- Haptoglobin (mg/dl) was marked by an immunoturbidimetric method according to Heidelberger and Kendall modified by Hellsing [28], based on the photometric measurement of specific insoluble immune complexes with a 340-nm wave; the total precision of the test is ≤3.3 % CV, and sensitivity is 0.26 g/l;
- Transferrin (mg/dl) was marked by an immunoturbidimetric method according to Heidelberger and Kendall modified by Hellsing [28], based on the photometric measurement of specific insoluble immune complexes with a 340-nm wave; the total precision of the test is ≤3.5 % CV, and sensitivity is 0.35 g/l;
- Fibrinogen (g/dl) was marked in the citrate plasma in accordance with a modified Clauss’ method [29, 30]; readings were conducted using a coagulometer manufactured by Behring Inc. (USA); the total precision of the test is ≤5.9 % CV, and sensitivity is 0.8 g/l.

### Cytokine Assays

In the serum, the following cytokines were marked: IL-1β, IL-6, and TNF-α.

Assays of the interleukins mentioned above were conducted by the immunoassay method, using ready tests manufactured by R&D (USA). Sensitivities of the methods are: 1.5 pg/ml (IL-1β), 2 pg/ml (IL-6), and 1.7 pg/ml (TNF-α). Readings were taken using a reader manufactured by BioTek (USA) EL×800.

### Statistical Analysis

The obtained values were subject to statistical analysis. Arithmetical means (*X*), standard deviations (SD), minimum value ranges (Min), and maximum value ranges (Max) were calculated. After prior verification of whether the calculated parameters were subject to normal decomposition (comparison of a histogram of variables with a graph of the Gaussian curve), the means of particular indices of the control group and the experimental inflammation group were compared using Student’s *t* test at the following levels of significance: 0.05, 0.01, and 0.001. The calculations were made using Statistica (v. 5.0).

## RESULTS

### Dynamics of Changes in Concentration of Serum Proteins in Rats: Results of Studies Conducted Using Electrophoresis

**Fig. 2:**
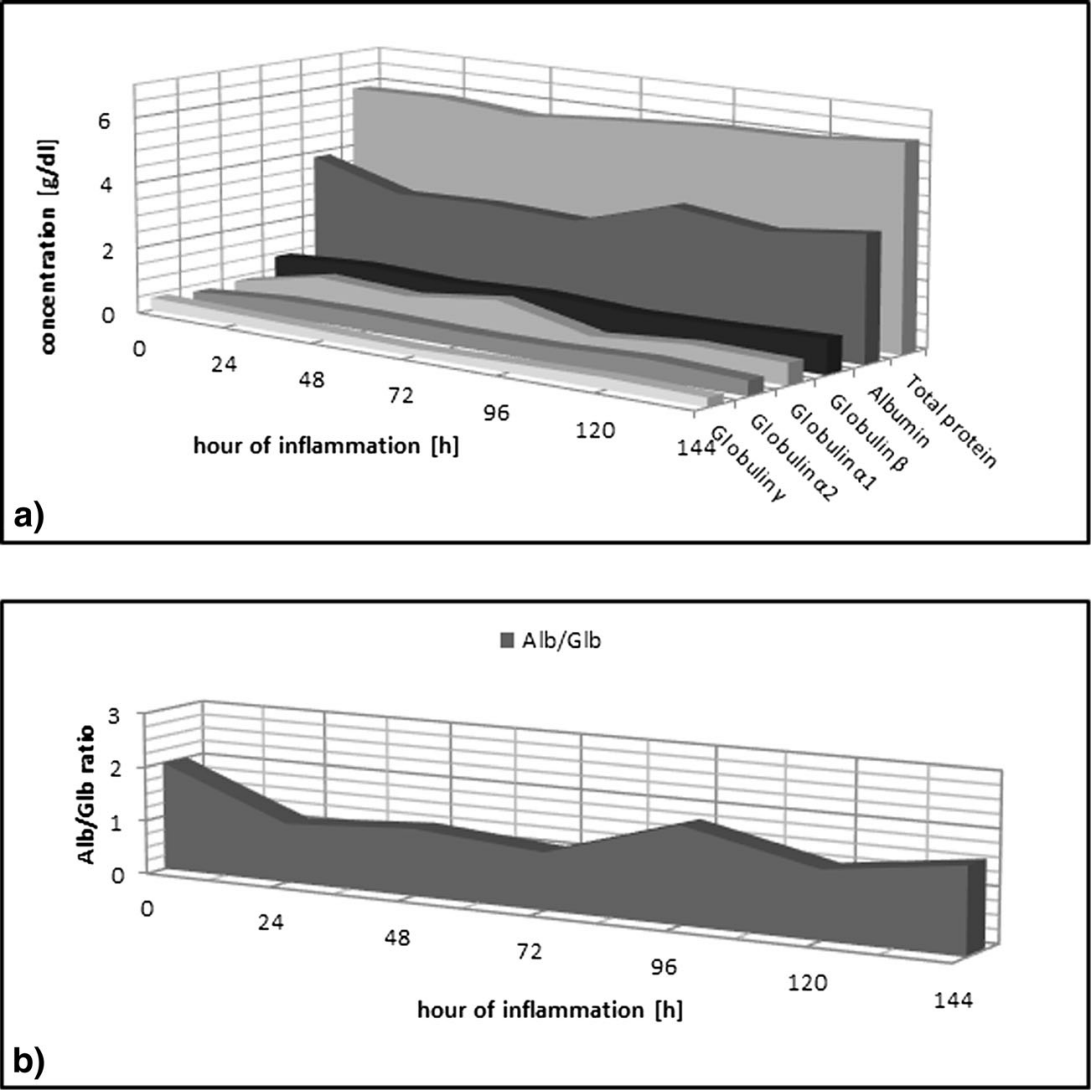
**a** Electrophoretic separation of rat serum proteins in induced pleurisy. **b** Changes of the albumin-to-globulin ratio during induced pleurisy.

At the 24th hour of inflammation, a significant reduction of albumin concentration is observed to be relative to the control value. The fraction of α_1_-globulin and α_2_-globulin increases significantly, which results in a change of the albumin/globulin (Alb/Glb) ratio (Table 1). The decrease of the albumin fraction at the 24th hour of inflammation described above is maintained (at the level of significant values) through the 72nd hour, when the maximum decrease of albumin concentration in the serum occurs, while simultaneously maintaining the decreased ratio of Alb/Glb. At subsequent time points (120 and 140 h), the increased concentration of the fraction of α_1_-globulin and α_2_-globulin and the decreased Alb/Glb ratio are maintained. There are no significant changes in β-globulin and γ-globulin fractions observed (Fig. 2).

**Table 1:** Electrophoretic Separation of Rat Serum in the Course of Experimental Pleurisy

		Albumin (g/dl)	Albumin (%)	Globulin								Total protein (g/dl)	Albumin/globulin
				α_1_ (g/dl)	α_1_ (%)	α_2_ (g/dl)	α_2_ (%)	β (g/dl)	β (%)	γ (g/dl)	γ (%)		
C	*N*	20	20	20	20	20	20	20	20	20	20	20	20
	*X*	3.84	66.16	0.374	6.5	0.303	5.24	0.896	15.38	0.391	6.72	5.81	2.01
	SD	0.48	5.06	0.14	2.58	0.106	1.92	0.181	2.31	0.122	1.93	0.54	0.42
IP (24 h)	*N*	5	5	5	5	5	5	5	5	5	5	5	5
	*X*	3.04	52.1	0.924	15.78	0.506	8.66	1.042	17.84	0.334	5.66	5.85	1.09
	SD	0.11	3.03	0.123	1.45	0.027	0.47	0.121	1.56	0.094	1.43	0.28	0.13
	*T*	0.001***	0.0000***	0.0000***	0.0000***	0.0003***	0.001***	0.104	0.035	0.343	0.267	0.875	0.0000***
IP (48 h)	*N*	5	5	5	5	5	5	5	5	5	5	5	5
	*X*	3.11	55.24	0.776	13.84	0.526	9.38	0.936	16.58	0.282	5.02	5.62	1.24
	SD	0.38	1.71	0.053	1.03	0.051	0.9	1.14	1.28	0.039	0.61	0.58	0.09
	*T*	0.005**	0.0000***	0.0000***	0.0000***	0.0001***	0.0000***	0.652	0.277	0.064	0.069	0.512	0.0000***
IP (72 h)	*N*	5	5	5	5	5	5	5	5	5	5	5	5
	*X*	2.99	51.32	1.108	19	0.462	7.86	1.048	17.98	0.226	3.86	5.83	1.05
	SD	0.09	1.72	0.021	0.41	0.085	1.43	0.051	0.85	0.065	1.05	0.11	0.07
	*T*	0.0008***	0.0000***	0.0000***	0.0000***	0.005**	0.009**	0.081	0.022*	0.008*	0.004**	0.923	0.0000***
IP (96 h)	*N*	5	5	5	5	5	5	5	5	5	5	5	5
	*X*	3.83	64.02	0.492	8.26	0.476	7.94	0.9	14.92	0.294	4.86	5.99	1.78
	SD	0.11	1.65	0.126	2.28	0.018	0.24	0.187	2.48	0.084	1.22	0.24	0.13
	*T*	0.96	0.367	0.101	0.177	0.002**	0.005**	0.965	0.701	0.109	0.054	0.468	0.243
IP (120 h)	*N*	5	5	5	5	5	5	5	5	5	5	5	5
	*X*	3.48	57.28	0.722	11.92	0.582	9.54	0.95	15.6	0.344	5.62	6	1.25
	SD	0.17	2.22	0.09	1.76	0.054	0.57	0.094	1.36	0.109	1.58	0.25	0.17
	*T*	0.112	0.0000***	0.0000***	0.0002***	0.0000***	0.0000***	0.531	0.838	0.441	0.255	0.441	0.001***
IP (144 h)	*N*	5	5	5	5	5	5	5	5	5	5	5	5
	*X*	3.8	61.04	0.652	10.48	0.454	7.32	1.052	16.92	0.27	4.28	6.23	1.57
	SD	0.21	2.35	0.115	2.03	0.085	1.51	0.127	1.84	0.094	1.47	0.17	0.16
	*T*	0.855	0.0000***	0.0004***	0.004**	0.007**	0.035	0.085	0.18	0.051	0.015*	0.101	0.032*

*C* control group, *IP* group with induced pleurisy, *N* number of animals, *X* arithmetic average, *SD* standard deviation, *T* statistical significance relative to control group (0.05 ≥ *T* > 0.01 − * | 0.01 ≥ *T* > 0.001 − ** | 0.001 ≥ *T* − ***)

### Activity of Liver Enzymes in Induced Pleurisy

#### Aspartate Aminotransferase

**Fig. 3:**
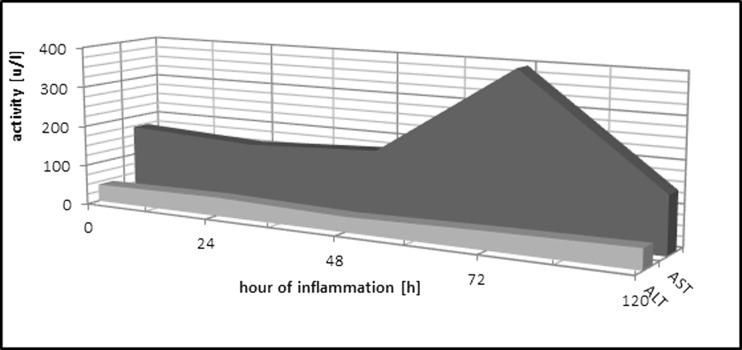
Changes in liver enzyme activities during induced pleurisy. *AST* aspartate aminotransferase, *ALT* alanine aminotransferase.

In the IP group, at the 72nd hour of inflammation, the activity of the enzyme increased significantly in comparison to the control group (Table 2, Fig. 3).

**Table 2:** Liver Enzymes in the Rat Serum in the Course of Experimental Pleurisy

		AST (U/l)	ALT (U/l)
C	*N*	10	21
	*X*	172.28	42.8
	SD	40.44	7.48
IP (24 h)	*N*	10	21
	*X*	156.22	46.28
	SD	37.57	9.24
	*T*	0.37	0.188
IP (48 h)	*N*	10	14
	*X*	172.32	37.65
	SD	48.99	8.46
	*T*	0.998	0.067
IP (72 h)	*N*	10	15
	*X*	394.44	44.71
	SD	28.59	6.59
	*T*	0.0000***	0.432

*C* control group, *IP* group with induced pleurisy, *AST* aspartate aminotransferase, *ALT* alanine aminotransferase *N* number of animals, *X* arithmetic average, *SD* standard deviation, *T* statistical significance relative to control group (0.05 ≥ *T* > 0.01 − * | 0.01 ≥ *T* > 0.001 − ** | 0.001 ≥ *T* − ***)

#### Alanine Aminotransferase

In the IP group, at the 72nd hour of inflammation, there were no significant changes in the activity of the enzyme observed in comparison to the control group (Table 2, Fig. 3).

### Analysis of Acute-Phase Proteins and Biochemical Indices in the Course of Experimental Pleurisy

#### C3 and C4 Complement Components

**Fig. 4:**
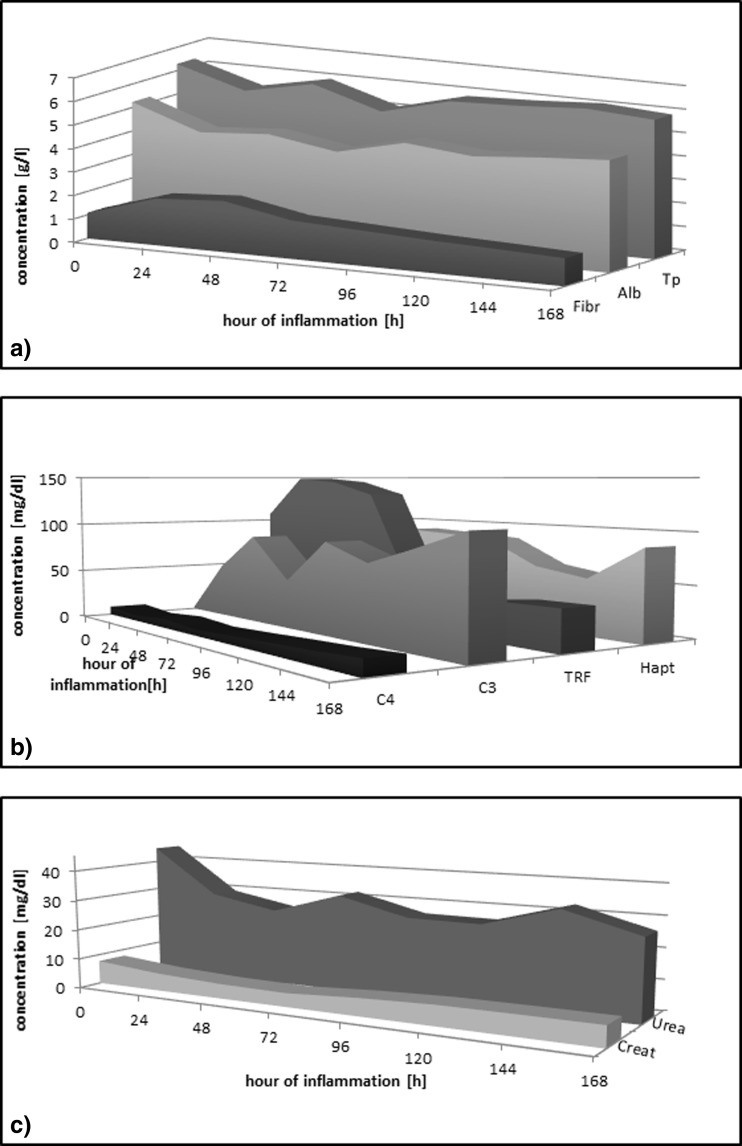
**a** Changes in the concentrations of albumin and total protein in the serum and fibrinogen in the rat plasma in the course of induced pleurisy. *Fibr* fibrinogen, *Alb* albumin, *Tp* total protein. **b** Changes in concentrations of biochemical parameters in the rat serum during induced pleurisy. *TRF* transferrin, *Hapt* haptoglobin, *C3* C3 complement protein, *C4* C4 complement protein. **c** Changes in levels of biochemical markers in induced pleurisy. *Creat* creatinine (concentration × 10^−1^).

An intensive increase of the C3 complement component was observed, starting from the 48th hour and continued through the 120th and 168th hours (Table 3). The C4 complement component indicated precarious stability at a low level for the first 3 days. From the 72nd hour of the inflammation, there was a successive, slow, and linear increase of the concentration of the protein (Fig. 4b).

**Table 3:** Biochemical Parameters of the Rat Serum and Plasma in the Course of Experimental Pleurisy

		Albumin (g/dl)	TP (g/dl)	TRF (mg/dl)	Haptoglobin (mg/dl)	Complement component		Urea (mg/dl)	Creatinine (mg/dl)	Fibrinogen (g/dl)
						C3 (mg/dl)	C4 (mg/dl)			
C	*N*	25	25	21	21	20	21	16	13	10
	*X*	5.24	6.29	106.74	4.91	1.79	8.43	44.84	0.772	1.09
	SD	0.35	0.69	10.24	2.43	0.82	2.09	6.99	0.099	0.2
IP (24 h)	*N*	29	31	24	25	24	24	16	13	7
	*X*	4.24	5.44	149.03	80.36	53.38	5.41	29.44	0.587	1.99
	SD	0.72	0.96	20.38	18.92	19.61	2.21	8.33	0.143	0.44
	*T*	0.0000***	0.0005***	0.0000***	0.0000***	0.0000***	0.0000***	0.0000***	0.0000***	0.0000***
IP (48 h)	*N*	23	28	23	23	19	23	14	14	11
	*X*	4.45	6.01	145.23	91.56	88.45	8.85	25.26	0.484	2.18
	SD	1.05	0.81	19.86	27.37	15.84	2.96	5.1	0.048	0.81
	*T*	0.0008***	0.179	0.0000***	0.0000***	0.0000***	0.596	0.0000***	0.0000***	0.0005***
IP (72 h)	*N*	26	31	26	26	22	24	15	15	6
	*X*	3.99	5.13	132.26	89.22	47.97	6	32.1	0.45	1.64
	SD	1.17	1.27	20.43	18.72	8.25	2.12	8.73	0.092	0.22
	*T*	0.0000***	0.0001***	0.0000***	0.0000***	0.0000***	0.0004	0.0000***	0.0000***	0.0002***
IP (96 h)	*N*	5	10	5	5	5	5	5	5	
	*X*	4.66	5.86	38.54	86.06	89.64	7.95	26.4	0.576	
	SD	0.24	0.47	1.89	9.74	8.19	1.61	3.05	0.053	
	*T*	0.002**	0.073	0.0000***	0.0000***	0.0000***	0.633	0.0000***	0.0008***	
IP (120 h)	*N*	5	10	5	5	5	5	5	5	15
	*X*	4.38	5.96	32.66	62.42	73.82	10.3	26.2	0.652	1.39
	SD	0.39	0.39	4.12	6.15	11.44	1.54	3.56	0.035	0.39
	*T*	0.0000***	0.156	0.0000***	0.0000***	0.0000***	0.074	0.0000***	0.019*	0.034*
IP (144 h)	*N*	5	10	5	5	5	5	5	5	
	*X*	4.62	6.13	38.76	55.04	90.44	12.29	32.8	0.66	
	SD	0.27	0.19	0.93	14	7.23	1.24	2.95	0.029	
	*T*	0.0009***	0.477	0.0000***	0.0002***	0.0000***	0.0007***	0.002**	0.027*	
IP (168 h)	*N*	5	5	5	5	5	5	5	5	
	*X*	4.8	5.94	39.76	87.86	108.26	15.25	26.34	0.655	
	SD	0.23	0.23	0.5	0.18	1.72	0.84	2.76	0.063	
	*T*	0.012*	0.27	0.0000***	0.0000***	0.0000***	0.0000***	0.0000***	0.027*	

*C* control group, *IP* group with induced pleurisy, *TP* total protein, *TRF* transferrin, *N* number of animals, *X* arithmetic average, *SD* standard deviation, *T* statistical significance relative to control group (0.05 ≥ *T* > 0.01 − * | 0.01 ≥ *T* > 0.001 − ** | 0.001 ≥ *T* − ***)

#### Transferrin

The shape of the curve of transferrin concentration changes in the serum in experimental inflammation is similar to a reversed letter S. Concentration changes of the protein relative to the control group at the monitored time points are statistically significant, and they constitute a significant decrease of concentration between the 72nd and 96th hours of inflammation (Fig. 4b).

#### Haptoglobin

Concentration of this indicative protein in the course of experimental pleurisy significantly increased at the 48th hour of the inflammation, after which it stabilized (plateau phase) and then slightly decreased between the 96th and 144th hours. From this time, an increase to its concentration was again observed (Fig. 4b).

#### Albumins and Total Protein

In the course of experimental pleurisy, it was observed that albumin and total protein concentrations in the serum in the physiological control group and the experimental inflammation group were maintained between the 24th and 168th hours of the process. A significant decrease of albumin concentration in the serum was observed, reaching a minimum value at the 72nd hour, while from the 120th to 168th hour, it showed an upward trend towards the physiological values. The inflammation process also significantly impacted the total protein concentration. The first decrease was observed at the 24th hour of the inflammation, while the second, significantly larger decrease appeared at the 72nd hour; from the 120th hour, an increase of concentration was observed (Fig. 4a).

#### Fibrinogen

Fibrinogen concentration in the process of experimental inflammation increased from the beginning of the process, peaking at the 48th hour of its duration, and after which, it decreased slightly up to the 72nd hour and then proceeded even slower up to the 168th hour of observation (Fig. 4a).

#### Urea and Creatinine

Urea concentration significantly decreased relative to the control values between the 24th and 48th hours of inflammation. Next, slight oscillations around the value were observed up to the 168th hour of inflammation. Creatinine concentration in the process was characterized by a dynamic and linear decrease, reaching its lowest value at the 72nd hour, and after which, it increased up to the 120th hour before stabilizing (plateau phase) (Fig. 4c).

### Analysis of the Level of Proinflammatory Cytokines in the Course of Experimental Pleurisy

#### Interleukin-1β

**Fig. 5:**
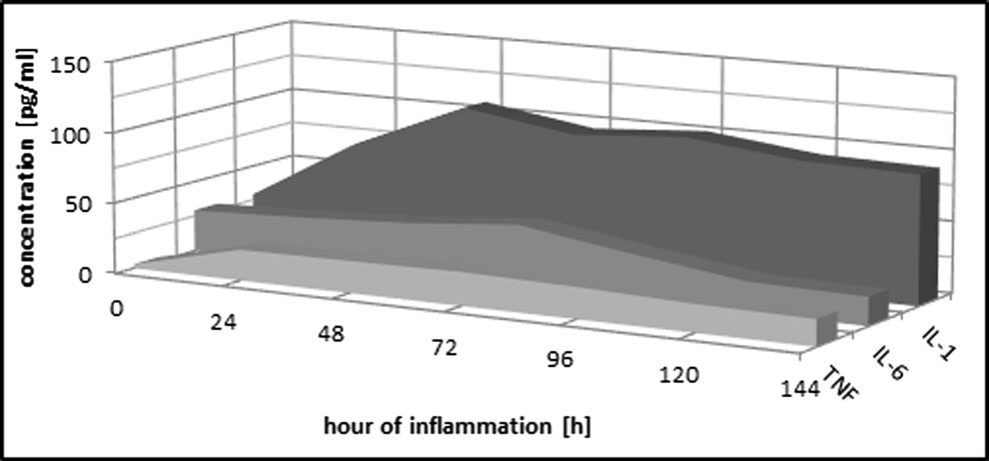
Changes in the concentrations of proinflammatory cytokines during induced pleurisy. *TNF* tumor necrosis factor, *IL-6* interleukin-6, *IL-1* interleukin-1β.

In the experimental pleurisy of rats, a statistically significant increase of this interleukin in the first 48 h of the process was observed, and after which, its concentration slightly fluctuated until the conclusion of observations around the value reached at the 72nd hour of inflammation (Table 4, Fig. 5).

**Table 4:** Proinflammatory Cytokines in the Rat Serum in the Course of Experimental Pleurisy

		IL-1 (pg/ml)	IL-6 (pg/ml)	TNF (pg/ml)
C	*N*	9	9	9
	*X*	29.12	29.23	2.9
	SD	6.73	10.58	1.09
IP (24 h)	*N*	6	7	6
	*X*	74.45	32.22	21.83
	SD	26.25	5.43	2.56
	*T*	0.0002***	0.509	0.0000***
IP (48 h)	*N*	6		6
	*X*	107.6		22.53
	SD	22.06		2.81
	*T*	0.0000***		0.0000***
IP (72 h)	*N*	6	7	6
	*X*	95.62	43.47	23.57
	SD	9.91	10.33	3.21
	*T*	0.0000***	0.017*	0.0000***
IP (96 h)	*N*	6		6
	*X*	101.18		22.08
	SD	14.87		2.59
	*T*	0.0000***		0.0000***
IP (120 h)	*N*	6	6	6
	*X*	92.23	21.34	19.55
	SD	7.71	5.02	0.99
	*T*	0.0000***	0.115	0.0000***
IP (144 h)	*N*	6		6
	*X*	90.6		18.52
	SD	20		1
	*T*	0.0000***		0.0000***

*C* control group, *IP* group with induced pleurisy, *IL-1* interleukin-1β, *IL-6* interleukin-6, *TNF* tumor necrosis factor, *N* number of animals, *X* arithmetic average, *SD* standard deviation, *T* statistical significance relative to control group (0.05 ≥ *T* > 0.01 − * | 0.01 ≥ *T* > 0.001 − ** | 0.001 ≥ *T* − ***)

#### Interleukin-6

Concentration of this interleukin maximally increased at the 72nd hour of inflammation, and then it significantly decreased at the 120th hour of its duration and maintained at the same level until the end of observations (Fig. 5).

#### TNF-α

Concentration of this index significantly increased in the first 24 h of inflammation and maintained its value almost without change for the entire duration of observations (Fig. 5).

## DISCUSSION

The results of the study of induced pleurisy in rats presented herein describe the dynamics of changes to protein fraction concentrations in the serum and plasma, and of selected acute-phase proteins, as well as nonspecific biochemical indices from the beginning of the process to the final hours of its duration. The findings are extraordinary and significant from the diagnostic and clinical perspective in view of the fact that the initial phases of inflammation (from its initiation to the 12th hour) cannot be diagnosed using laboratory indices, despite an existing pain reaction and local changes in temperature (thermal vision). After the first phase of inflammation, lasting several hours, a decrease of albumin concentration occurs, which is indicated by studies conducted using both electrophoresis as well as the colorimetric method. Albumin is a protein of the negative acute phase [18, 21–23, 31, 32], which means that a decrease in its concentration causes a decrease in the Alb/Glb ratio. The effect of these changes during this phase of inflammation is quicker erythrocyte sedimentation, which consequently results in an increase of the ESR value. The percentage decline of albumin concentration and the decreased Alb/Glb ratio are observed up to the 144th hour of inflammation. However, from the 96th hour, there is a slight increase in the latter index. Analyzing the quality and quantity of the composition of fractions of α_1_-globulin, α_2_-globulin, β-globulin, and γ-globulin during the course of inflammation, it can be stated that from the beginning to the 72nd hour, a significant increase of fractions of α_1_-globulin and α_2_-globulin occurs. Increased concentration of the fraction of α_1_-globulin persists up until the 144th hour of inflammation. Studies using electrophoresis indicate that during the inflammation process, there are no significant changes in fractions of β-globulin and γ-globulin, and only at the 72nd hour, a slight decrease of γ-globulin concentration is observed.

Fibrinogen is an acute-phase protein which reacts to inflammation with increased concentration. Our studies also indicated that a significant increase of fibrinogen concentration occurs during the first 3 days of inflammation. Other authors report that this process was observed during the first 48 h of inflammation [18, 32].

IL-6, released by monocytes and macrophages, leads to an increase of hepatocyte activity in inflammation, which, in turn, results in an increase of fibrinogen synthesis [33]. Factors which stimulate this process include products of fibrin and fibrinogen degradation occurring as a result from plasmin, and protease activity occurring in inflammation [33–36]. This process compensates for losses in fibrinogen used in the inflammatory reaction. It is accompanied by the phenomenon of local disseminated intravascular coagulation (DIC), within which a significant decrease of the number of platelets and procalcitonin (PCT) and platelet distribution width (PDW) indices occurs at the 72nd hour of induced inflammation. This phenomenon was also observed in our previous studies in a histopathological image of a lung patch [7, 37].

Haptoglobin is a protein included in the group of positive reactants and in the first group of proteins stimulated by IL-1 [20, 38, 39]. In inflammatory conditions and conditions of tissue damage, its concentration in the serum increases within 48 h and then returns to normal within 7 days [35, 40]. Haptoglobin prevents the loss of iron [41–43]. Hemolysis of erythrocytes related to the activation of the complement, which accompanies injuries, leads to decreased iron concentrations in the body. Free haptoglobin causes oxidation of arachidonic acid and oxidation of lipids in the membranes of erythrocytes, causing their hemolysis [44]. Haptoglobin stoichiometrically and permanently binds hemoglobin from decomposed erythrocytes [27, 42, 45]. Our study indicated that together with a decrease of hematological indices between the 48th and 120th hours of inflammation [9], at the beginning of the phase, there is a 19-fold increase of haptoglobin, which persists at a stable level up to the 96th hour. Transferrin, which also participates in the protection of hemoglobin iron, is included in the group of negatively reactive proteins, *i.e.*, reacting to inflammation with decreased concentration [31, 46]. However, our observations proved that during the first hours of induced inflammation, paradoxically, a significant increase of this index is observed (by 37 %), which persists up to the 72nd hour; next, a rapid decrease of its concentration by 70 % occurs in relation to the control value. The increase can be explained by the synthesis of the protein induced through decomposition of erythrocytes in the inflammatory process. A 49-fold increase of the C3 complement component observed at the 48th hour of inflammation is a contributing factor, as it correlates with a decrease of red blood cells and hemoglobin persisting up to the 168th hour. Interestingly, the C4 complement component, which is also an acute-phase protein contributing to erythrocyte hemolysis, responds to inflammation by doubling its concentration quite late, at the 144th–168th hours of the process. In the time interval between the 48th and 96th hours of inflammation, an increase of inflammatory reaction intensity occurs, which is reflected by the observed dynamics of changes of inflammation markers (haptoglobin, C3 and C4 complement components). The changes of these indices described above reach their peak between the 72nd and 96th hours of pleurisy, which correlates with the described local changes of temperature in induced pleurisy in rats [1]. Observations conducted within our previous studies regarding the behavior of collagen degradation products in experimental pleurisy [47–49] confirm the above observations. Studies conducted by other authors on a similar experimental model, but taking into account other biochemical indices, indicated that the period between the 48th and 96th hours of inflammation plays an important role in restoring homeostasis in the body [50].

In our studies, we also analyzed nonspecific biochemical indices which are not used to assess inflammation, yet which do show the status of biochemical changes in the body during various disease processes. These indices include AST and ALT which constitute indicative enzymes for such diseases as liver damage. By monitoring the status of damage to the organ using the aminotransferases mentioned above, it is possible to assess its capacity for synthesis of serum and plasma proteins, which are important in inflammatory processes. Liver damage disrupts the course of inflammation [49, 51]. Our studies of the course of inflammation revealed a significant increase in the activity of aspartate aminotransferase (greater than twofold) occurring on the third day of inflammation. No significant changes in the activity of alanine aminotransferase were observed. The increase of AST is explained by the liver’s quick response resulting from its enhanced metabolism in inflammation and distribution of inflammation in the lungs, which has been observed by other authors [19]. The inflammation process generates a negative nitrogen balance [52, 53], which is associated with a focally increased metabolism and the systemic effects of glucocorticoids, whose production rises in inflammation as well as due to the destructive effect of TNF and fever. Locally, inflammation can manifest itself in the destruction of inflammatory tissue [26, 47]; more generally, it is related to catabolic changes in proteins, primarily albumins in the liver, which is manifested in a decrease of their concentration in the serum [21, 32, 34, 54] and potential increases in concentrations of the products of protein metabolism, such as urea and creatinine in the serum [19].

The results presented in this paper prove that in the course of experimental inflammation, concentrations of urea and creatinine in the blood serum decrease at the 72nd hour of the process. This fact is quite difficult to explain, considering the data existing in the literature. It is suspected that the increased elimination of the two metabolites with urine occurs as a result of intensified diuresis or that it results from decreased liver function [19]. It is said that kinins play a significant role in protein metabolism occurring as a part of the inflammatory process. Some authors observed intensified synthesis of proteins in skeletal muscles as a result of kinins [55, 56]. This effect of their anabolic activity on protein metabolism has been observed in postoperative patients [57].

Our study of the course of experimental pleurisy revealed a rapid (7.5-fold) increase of TNF-α concentration at the 24th hour of inflammation. Interleukin-1β is another proinflammatory cytokine which responds with a significant increase in concentration during the first hours of inflammation, specifically by a factor of 3.5. The lowest increase of concentration in comparison to the interleukins listed above (1.5-fold) was observed in respect to IL-6 concentration, and it occurred during the later hours of inflammation. Other authors report an effect of IL-1β on the production of interleukin-1 receptor accessory protein (IL-1RAP), observed in infections and inflammatory reactions a rather short time after the activation of the inflammatory factor [52, 58]. TNF-α and interferon (IFN)-γ also contribute significantly to the release of IL-1RAP, which is related to IL-1 receptors, but which does not result in their activation. It may cause a decrease in inflammatory reaction and sensitivity to pain in inflammation. Moreover, the decreased vascular component, resulting from the impact of IL-1 on endothelial cells and inhibition of elastase secretion by neutrophils, is related to IL-1RAP blocking the activity of IL-1 [58]. According to some authors [59], there is a correlation between IL-6, produced by activated monocytes and macrophages in the focal of inflammation, and the concentration of acute-phase proteins produced by hepatocytes stimulated by the interleukin. IL-6 also plays an important role in stimulation of thrombocytopoiesis, which contributes to complementing the losses of platelets in the focal of inflammation. Stimulation of the hypothalamic-pituitary axis and secretion of cortisol, as a result of ACTH, inhibits the synthesis of proinflammatory cytokines and the release of IL-1RAP, which is alleviated by IL-4 [52, 60, 61]. Analyzing the results of our study, including interleukin concentration, it can be stated that TNF-α plays an important role in inflammatory reactions while IL-1 is less significant. It may constitute a reason for releasing a smaller number of the antagonist of IL-1, which is RAP. It does not seem, however, that the significance of IL-6 in proportional synthesis of acute-phase proteins has found a definite and clear confirmation in the results we have obtained.

## CONCLUSIONS

Studying the course of pleurisy in rats, significant changes in biochemical indices were observed between the 48th and 96th hours of inflammation. A decrease of albumin concentration as well as a decrease of transferrin, urea, and creatinine concentrations were observed. On the other hand, C3 and C4 complement components increased, as did haptoglobin and fibrinogen concentrations. An early increase of IL-1 was also observed, and later on, increases of IL-6 and TNF were noted as well.

The processes mentioned above, reaching their maximum intensity between the 72nd and 96th hours of inflammation, correlate with previously described changes in the hematological image of blood and the characteristics of induced pleurisy identified on the basis of the results of tests performed using thermal vision [1, 9].
